# Supplementary material for: Diverse Sulfuriferula spp. from sulfide mineral weathering environments oxidize ferrous iron and reduced inorganic sulfur compounds
Source: Appl Environ Microbiol. 2025 Jun 5;91(7):e00216-25. doi: 10.1128/aem.00216-25 (PMC12285272; doi:10.1128/aem.00216-25)
Supplement: Supplemental material — Table S1; Fig. S1 and S2. [file aem.00216-25-s0001.pdf]

Supplementary Figures and Tables

Diverse *Sulfuriferula* spp. from sulfide mineral weathering environments oxidize ferrous iron- and reduced inorganic sulfur compounds

Kathryn K. Hobart[1,2], Gabriel M. Walker[1], Joshua M. Feinberg[1,2], Jake V. Bailey[1], Daniel S. Jones[1,3,4]

[1]Dept. Of Earth & Environmental Sciences, University of Minnesota, Minneapolis, MN 55455, U.S.A.
[2]Institute for Rock Magnetism, University of Minnesota, Minneapolis, MN 55455, U.S.A.
[3]Dept. of Earth and Environmental Science, New Mexico Institute of Mining and Technology, Socorro, NM 87801, U.S.A
[4]National Cave and Karst Research Institute, Carlsbad, NM, 88220, U.S.A.

Correspondence: Kathryn Hobart (khobart@usgs.gov), Daniel Jones (daniel.s.jones@nmt.edu)

Current addresses:
Kathryn K. Hobart: United States Geological Survey, Geology, Energy, and Minerals Science Center, Reston, VA, USA
Gabriel M. Walker: Diagnostic Radiology Residency, Henry Ford Hospital, Detroit, MI

Supplementary Table S1. Genome sizes and growth characteristics of strains of *Sulfuriferula* spp. described in this study and other named strains of *Sulfuriferula*. + indicates that growth was observed, - indicates growth was not observed, and blank cells indicate a substrate that was not tested.

	S. multivorans TTN	*S. plumbiphila* Gro7	*S. thiophila* mst6	*S. nivalis* SGTM	*S.* sp. strain AH1	*S.* sp. strain GW1	*S.* sp. strain GW6	*S.* sp. strain HF6a
Genome Size	3,619,383	3,452,774	2,834,181	3,361,892	**2,916,145**	**3,363,341**	**3,363,341**	**3,329,881**
pH range	5.3 – 8.6	4.0 – 7.8	4.6 – 8.1	4.3 – 7.4	**4.5 – 8***	**5 – 8***	**6 – 8***	**4 - 7**
Growth on H^2	-	+	-	-	**-**	**-**	**-**	**-**
Growth on sulfide (H_2S)	-	+	-	-	+	+	+	+
Growth on thiosulfate ($S_2O_3^2$-)	+	+	+	+	+	+	+	+
Growth on tetrathionate ($S_4O_6^{2-}$)	+	+	+	+	**-**	**-**	**-**	**-**
Growth on sulfite ($SO3^{2-}$)					**-**	**-**	**-**	**-**
Growth on elemental sulfur (S^0)	+	+	+	+	**-**	**-**	**-**	**-**
Growth on Fe^{2+}					+	+	+	+
Heterotrophic growth	+	-	-	-	**-**	**-**	**-**	**-**
Nitrate reduction	+	-	-	-	**-**	**-**	**-**	**-**

Bold are strains described in this study.
* pH above 8 not tested

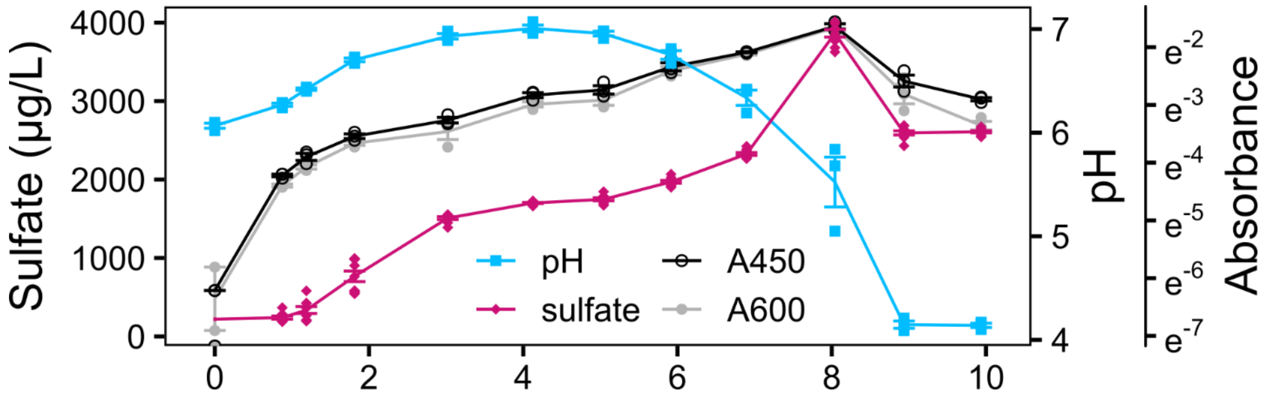

Supplementary Figure S1. pH, aqueous sulfate concentration, and absorbance at 450nm (A450) and 600nm (A600) for *Sulfuriferula* sp. strain AH1 grown on thiosulfate in pH 6.0 growth media.

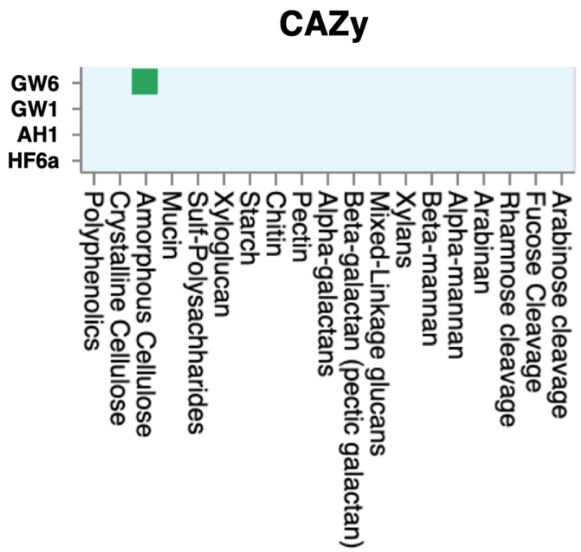

Supplementary Figure S2. CAZy annotations for the four strains characterized here, based on DRAM annotations. The heatmap shows presence (green) or absence (pale blue-white) of genes for cleavage of that carbohydrate category.
